# Gene Expression Profiling of Markers of Inflammation, Angiogenesis, Coagulation and Fibrinolysis in Patients with Coronary Artery Disease with Very High Lipoprotein(a) Levels Treated with PCSK9 Inhibitors

**DOI:** 10.3390/jcdd9070211

**Published:** 2022-07-01

**Authors:** Katja Hrovat, Andreja Rehberger Likozar, Janja Zupan, Miran Šebeštjen

**Affiliations:** 1 Biotechnical Faculty, University of Ljubljana, Jamnikarjeva 101, 1000 Ljubljana, Slovenia; kh5984@student.uni-lj.si; 2 Department of Vascular Diseases, University Medical Centre Ljubljana, Zaloška cesta 7, 1000 Ljubljana, Slovenia; andreja.rehbergerlikozar@kclj.si (A.R.L.); miran.sebestjen@guest.arnes.si (M.Š.); 3 Department of Clinical Biochemistry, Faculty of Pharmacy, University of Ljubljana, Aškerčeva 7, 1000 Ljubljana, Slovenia; 4 Department of Cardiology, University Medical Centre Ljubljana, Zaloška cesta 7, 1000 Ljubljana, Slovenia; 5 Department of Internal Medicine, Faculty of Medicine, University of Ljubljana, Korytkova ulica 2, 1000 Ljubljana, Slovenia

**Keywords:** inflammation, coagulation, fibrinolysis, lipoprotein(a), PCSK9 inhibitors, coronary artery disease, interleukin-1, vascular endothelial factor-A, tissue factor, plasminogen activator inhibitor-1

## Abstract

Besides lipids, inflammation, angiogenesis, coagulation and fibrinolysis play very important roles in coronary artery disease (CAD). We measured gene expression of the inflammatory markers interleukin (IL)-1β (*IL1B*) and interferon (IFN)-γ (*IFNG*), vascular endothelial growth factor-A (VEGF-A) (*VEGFA*), and coagulation and fibrinolysis markers tissue factor (TF) (*F3*) and plasminogen activator inhibitor-1 (PAI-1) (*SERPINE*) in healthy controls and CAD patients with high lipoprotein(a) (Lp(a)). The aim of our study was to identify, first, if there is a difference in these markers between controls and patients; secondly, if these markers are associated with lipids; and third, what the influence of proprotein convertase subtilisin/kexin type 9 (PCSK9) inhibitors is on these markers. We included 124 subjects, 27 controls and 97 patients with CAD (30 in placebo and 67 in the PCSK9 group). Blood samples were collected for lipid and gene measurement. The results showed higher expression of *IL1B* (*p* < 0.0001), *VEGFA* (*p* < 0.0001), and *F3* (*p* = 0.018) in controls in comparison with patients. Significant correlations were observed between *IL1B* and lipids. Treatment with PCSK9 inhibitors increased *VEGFA* (*p* < 0.0001) and *F3* (*p* = 0.001), and decreased *SERPINE* (*p* = 0.043). The results of our study underpin the importance of IL-1β, VEGF-A and TF in CAD as well as the effect of PCSK9 treatment on these markers.

## 1. Introduction

Despite highly effective lipid-lowering drugs, high systemic lipid levels remain a major risk factor in recurrent acute cardiovascular events [1]. Proprotein convertase subtilisin/kexin type 9 (PCSK9) inhibitors lower low-density lipoprotein (LDL) cholesterol by more than 50% in patients treated with the highest tolerated dose of statin, while also reducing lipoprotein(a) (Lp(a)) by 20–40% [2]. Lp(a) is an independent risk factor for acute cardiovascular events regardless of LDL cholesterol levels [3]. Both LDL cholesterol and Lp(a) cholesterol are associated with inflammation, coagulation and fibrinolysis, which are important in all stages of the atherosclerotic process [4]. Interferon (IFN)-γ is a classic representative of inflammatory mediators expressed at high levels in atherosclerotic lesions [5] that stimulates an inflammatory response in vascular smooth muscle cells. Interleukin (IL)-1β is important as a local vascular and systemic contributor of inflammation in the atherosclerotic process. IL-1β acts on vascular endothelial and smooth muscle cells and macrophages to produce other proinflammatory cytokines such as IL-6, which stimulates the production of C reactive protein (CRP), which predict first-ever and recurrent cardiovascular events beyond those predicted by conventional risk algorithms [6]. Vascular endothelial growth factor-A (VEGF-A), a key mediator of angiogenesis, was increased in patients after myocardial infarction and correlated with high concentrations of inflammatory cytokines [7]. Hence, these data suggest that increased levels of VEGF-A are part of ongoing inflammatory activity. Since high concentrations of VEGF-A in patients with myocardial infarction lead to neovascularization of the inflamed plaques and their destabilization, the VEGF-A plasma levels provide a negative prognostic value.

The coagulation process is stimulated by the damage to the vascular wall. At the same time, blood comes into contact with tissue factor (TF), which triggers the coagulation process via the TF/VIIa complex [8,9]. The result of the coagulation process is the formation of thrombin, which converts soluble fibrinogen into insoluble fibrin, which is the basis of blood clots [10]. The synthesis of TF in endothelial cells represents the strongest procoagulant mechanism by which endothelial cells participate in homeostasis. On the other hand, cells that synthesize and contain TF have been found in atherosclerotic beds. These cells presented macrophages in the form of foam cells, monocytes along cholesterol lines, as well as mesenchymal cells in the intima of the arterial wall [11]. It is not entirely clear whether macrophages synthesize TF themselves or whether it originates from phagocytosed cell debris found in the necrotic cortex of the atherosclerotic bed.

The fibrinolytic system degrades fibrin through plasminogen-derived plasmin in a series of reactions [12]. The main factors regulating the activity of the fibrinolytic system are tissue plasminogen activator (t-PA), which accelerates the fibrinolytic process, and the tissue plasminogen activator inhibitor (PAI-1), which inhibits it [13]. Both t-PA and PAI-1, are secreted by endothelial cells [14]. PAI-1 is the most important inhibitor of t-PA and the balance of both molecules regulates the functioning of the fibrinolytic process [15].

Treatment with statins significantly decreases markers of inflammation [16] and has favourable effects on markers of coagulation and fibrinolysis [17]. PCSK9 inhibitors have shown no influence on high sensitivity CRP (hsCRP) levels regardless of the PCSK9 inhibitor type, patient characteristics, concomitant treatment or treatment duration [18]. No data are available about the effects of the PCSK9 inhibitors on markers of coagulation and fibrinolysis.

Since the markers of coagulation, fibrinolysis, inflammation and angiogenesis play a very important role in the atherosclerotic process, the aim of our study was to compare the gene expression of these markers in patients with coronary artery disease (CAD) with very high levels of Lp(a) and healthy controls, and to evaluate the effect of PCSK9 treatment on these markers in CAD patients.

## 2. Materials and Methods

### 2.1. Study Subjects

A total of 124 subjects (27 healthy controls and 97 patients with CAD) were included in our study. Healthy controls were adults with no history or risk factors for CAD. The patients had clinically stable CAD for at least six months after myocardial infarction, which occurred before the age of 50 years. Only patients with an Lp(a) level >1000 mg/L or Lp(a) level >600 mg/L and LDL-cholesterol level >2.6 mmol/L were included in the study. All patients received maximally tolerated statin therapy and ezetimibe if needed, including β-blockers, antiplatelet drugs, acetylsalicylic acid and angiotensin-converting enzyme inhibitors. Their therapy had not been changed for at least two months before enrollment in the study. The main exclusion criteria were liver transaminases elevated more than three times above reference values, severe renal dysfunction, serum creatinine level higher than 200 mmol/L, and acute illness in the previous six weeks.

After enrolment, all patients underwent clinical and laboratory evaluation and were then randomized into two groups, as described before [19]. The PCSK9 inhibitor treated group (N = 67 patients) received a PCSK9 inhibitor, i.e., alirocumab (150 mg, N = 34 patients) or evolocumab (140 mg, N = 33 patients), subcutaneously, every two weeks, whereas the second group received placebo (N = 30 patients). The sample size was determined based on the 0.80 power of the study, 0.15 effect size and 0.05 α error probability using Gpower [20]. Clinical and laboratory parameters were measured in both placebo and PCSK9 inhibitor treated groups, at the beginning of the study and after six months of placebo or the PCSK9 inhibitor treatment period, as previously described [19].

The study protocol was approved by the Slovenian Ethics Committee for Research in Medicine (0120-357/2018/8). All participants gave written informed consent in accordance with the Declaration of Helsinki.

### 2.2. Blood Collection and Lipid Profile Determination

Peripheral blood samples from all subjects were collected in tubes without and with anticoagulant (K3-EDTA) to obtain serum and plasma for biochemical analyses. All lipid parameters were measured in the Laboratory of Haemostasis and Atherothrombosis at the Clinical Department of Angiology, University Clinical Centre Ljubljana, according to standard laboratory procedures. An aliquot of peripheral blood (3 mL) was collected for RNA isolation and gene expression measurement at the beginning of the study and after six months of placebo or PCSK9 inhibitor treatment period in Tempus™ Blood RNA Tube (Thermo Fisher Scientific, Waltham, MA, USA), well mixed and stored at −80 °C until further processing at the Department of Clinical Biochemistry, Faculty of Pharmacy, University of Ljubljana.

### 2.3. Total RNA Isolation and Evaluation

Total RNA was extracted from the samples collected in Tempus™ Blood RNA Tubes using a Tempus™ Spin RNA Isolation Kit (Thermo Fisher Scientific, Waltham, MA, USA) according to the manufacturer’s instructions. The concentration and purity of the isolated RNA was determined using a NanoDrop™ One/OneC Microvolume UV-Vis Spectrophotometer (Thermo Fisher Scientific, Waltham, MA, USA). Complementary DNA (cDNA) was synthesized using a High-Capacity cDNA Archive kit (Thermo Fisher Scientific), according to the manufacturer’s instructions, and a peqSTAR thermal cycler (VWR, Radnor, PA, USA).

### 2.4. Gene Expression Profiling

Gene expression measurements were performed according to the MIQE guidelines [21]. Quantitative polymerase chain reaction (qPCR) was performed using 5× HOT FIREPol EvaGreen qPCR Supermix (Solis BioDyne, Tartu, Estonia) according to the manufacturer’s protocol and LightCycler 480 II instrument (Roche, Basel, Switzerland). Specific conditions were as follows: initial activation at 95 °C 12 min (1 cycle), denaturation at 95 °C 15 s, annealing at 60 °C 30 s, and extension at 72 °C 30 s (all three steps 45 cycles), melting curve formation at 65–97 °C (one cycle). The sequences of the primers (Sigma-Aldrich, St. Louis, Missouri, United States) for genes *IL1B*, *IFNG* and *VEGFA* (Supplementary Table S1) were used from previous studies [22,23,24]. The sequences of the primers for genes *F3* and *SERPINE* (Supplementary Table S1) were obtained from online sources [25,26] and validated on our samples. The gene expression data were obtained using the standard curve and the second derivative maximum method (LightCycler 480 software 1.5.0, Roche, Basel, Switzerland). All of the data were normalized to geometric mean of glyceraldehyde-3-phosphate dehydrogenase (*GAPDH*) and ribosomal protein L13a (*RPL13A*).

### 2.5. Statistical Analysis

**Figure 1:**
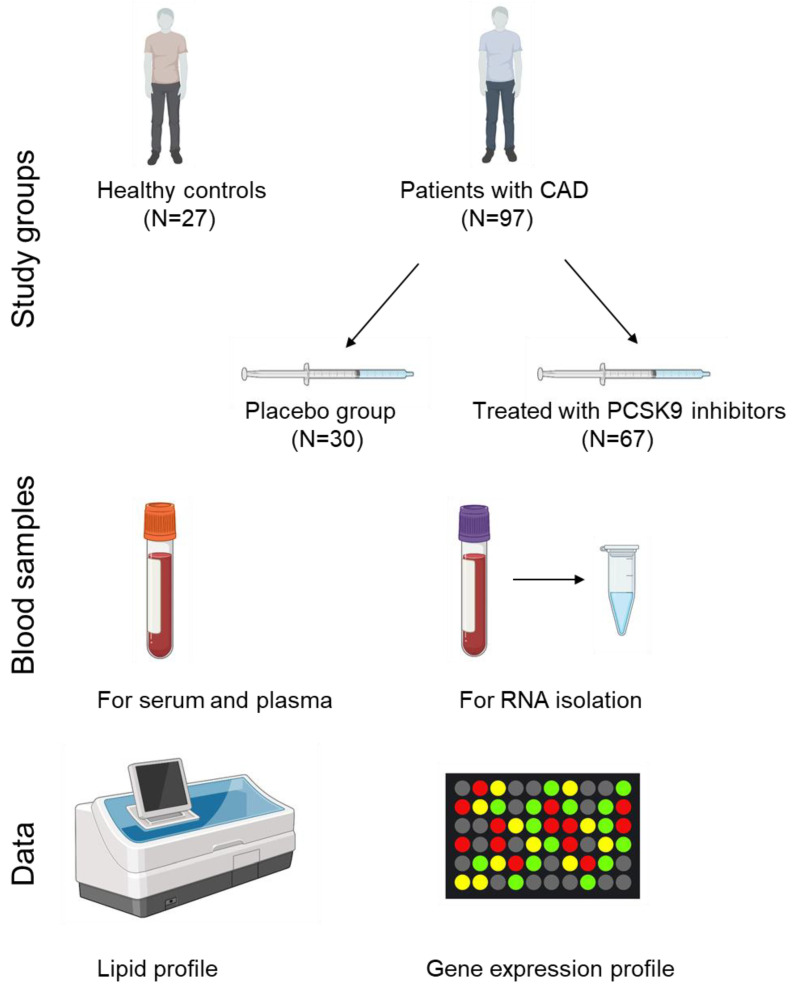
The study design. Subjects included in the study were healthy adults with no history of CAD and patients with CAD. Patients with CAD were randomized to the placebo group and the group treated with PCSK9 inhibitors, i.e., alirocumab (150 mg, N = 34 patients) or evolocumab (140 mg, N = 33 patients) for six months. Blood samples were taken at a single point for healthy controls and at the baseline and after six months of treatment for CAD patients. The lipid profile was measured and gene expression for the five studied genes (*IL1B*, *IFNG*, *VEGFA*, *F3*, *SERPINE*), and two reference genes (*GAPDH* and *RPL13A*) was determined.

The normality of the distribution of the data was tested using a Kolmogorov-Smirnov test. Since the data were not normally distributed, non-parametric tests were used. To compare the data between the controls and the patients, a Mann-Whitney U test was used. To compare the basic characteristics between the three study groups, namely healthy controls, placebo and treated groups, an independent samples Kruskal-Wallis test with a Bonferroni multiple comparison test was used. To compare the related samples data, i.e., the gene expression before and after treatment within the placebo and the treated groups, a Wilcoxon signed rank test was used. For correlation analysis between the lipid profile parameters and the gene expression data, a Spearman correlation analysis was used. The statistical analyses were performed with IBM SPSS Statistics version 27 and Graph Pad Prism version 8.4.3 for Windows (GraphPad Software, San Diego, CA, USA. *p* values < 0.05 were considered as statistically significant. The study design is summarized in Figure 1. Figure 1 was created using BioRender.com (accessed on 26 May 2022).

## 3. Results

### 3.1. Study Groups

A total of 124 subjects were included in the study. The subjects were divided into three groups, namely healthy controls, patients with CAD treated with placebo, and patients with CAD treated with PCSK9 inhibitor (alirocumab or evolocumab). Baseline characteristics and lipid profile of the subject groups are shown in Table 1. Based on the inclusion criteria and the nature of the CAD, significantly more male patients were included in our study. However, there were no differences in age (*p* = 0.108, Kruskal-Wallis test) and male/female ratio (*p* = 0.103, Chi-squared test) between the three studied groups. The healthy controls had statistically significantly higher lipid parameters, namely total, HDL and LDL cholesterol, apolipoprotein A1 and B levels in comparison with the placebo group (Kruskal-Wallis test with Bonferroni multiple comparison tests). The healthy controls also showed statistically significantly higher lipid parameters, namely total, HDL and LDL cholesterol, apolipoprotein A1 and B levels in comparison with the PCSK9 inhibitor treated group (Kruskal-Wallis test with Bonferroni multiple comparison tests). The levels of triglycerides were not significantly different between the groups (*p* = 0.853, Kruskal-Wallis test). There were no statistically significant differences in any of the baseline characteristics between the placebo and the PCSK9 inhibitor treated groups (*p* > 0.05, Kruskal-Wallis test with Bonferroni multiple comparison tests).

### 3.2. Healthy Controls Show Higher Expression of IL1B, VEGFA and F3 in Comparison with CAD Patients

**Figure 2:**
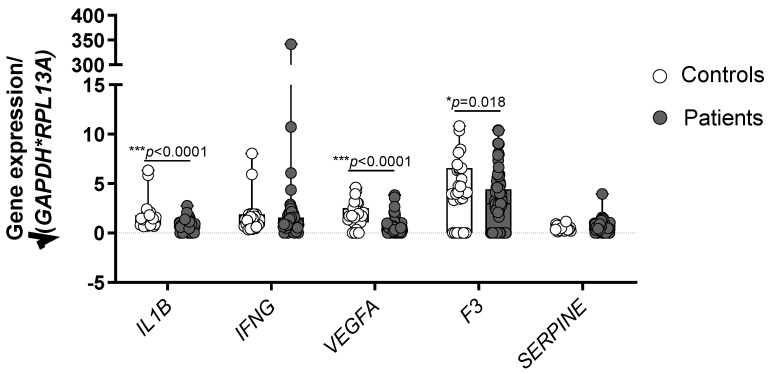
Comparison of the gene expression profile between healthy controls and patients with CAD. Significant differences were obtained for *IL1B*, *VEGFA* and *F3* as indicated (*** *p* < 0.0001, * *p* < 0.05, Mann Whitney U test). Shown are box and whisker plots with individual values. Gene expression data are normalized to geometric mean of glyceraldehyde-3-phosphate dehydrogenase (*GAPDH*) and ribosomal protein L13a (*RPL13A*).

To determine if the studied markers have a role in CAD, their gene expression profile was compared between the healthy subjects and patients with CAD (Figure 2). Statistically significant results were obtained for *IL1B* (*p* < 0.0001), *VEGFA* (*p* < 0.0001), and *F3* (*p* = 0.018). Interestingly, healthy controls showed higher levels of all statistically significant genes. The results for *IFNG* and *SERPINE* showed no statistically significant differences (*p* = 0.319 and 0.344, Mann-Whitney U test).

### 3.3. Significant Correlations between IL1B and SERPINE Expression and Lipid Parameters in Controls and CAD Patients

**Figure 3:**
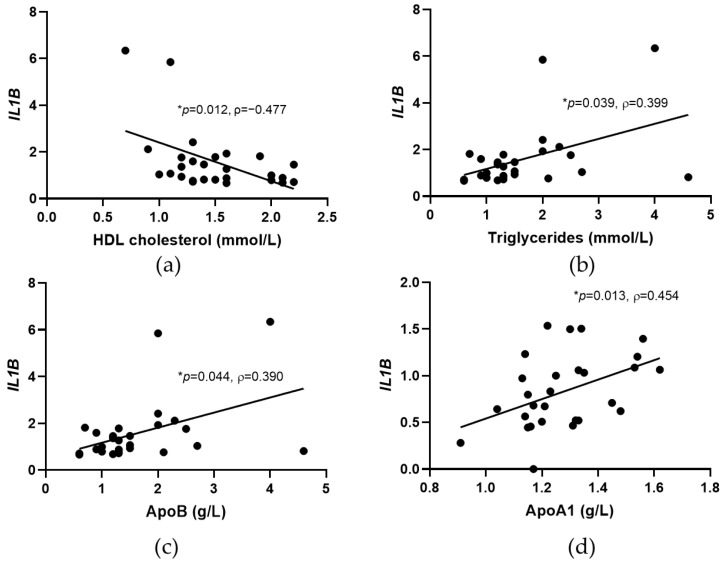
Significant correlations between *IL1B* expression and lipid parameters. Significant correlations were found between *IL1B* and HDL cholesterol (**a**), triglyceride levels (**b**), and apolipoprotein B (ApoB) (**c**) in healthy controls, and between *IL1B* and apolipoprotein A1 (ApoA1) (**d**) in patients with CAD prior treatment with PCSK9 inhibitors. Scatter plots are shown with the results of Spearman correlation analysis (* *p* < 0.05; ρ, Spearman’s correlation coefficient). Gene expression data are normalized to geometric mean of glyceraldehyde-3-phosphate dehydrogenase (*GAPDH*) and ribosomal protein L13a (*RPL13A*).

To evaluate if the gene expression profile of the studied markers is associated with systemic lipid parameters, a Spearman correlation analysis was performed in healthy controls and patients with CAD before and after PCSK9 inhibitor treatment. In the healthy control group, significant negative correlation was observed for *IL1B* and HDL cholesterol (ρ = −0.477, *p* = 0.012), and positive correlations between *IL1B* and triglycerides (ρ = 0.399, *p* = 0.039), and apolipoprotein B levels (ApoB) (ρ = 0.390, *p* = 0.044), as shown in Figure 3. In patients with CAD, a prior PCSK9 treatment significant positive correlation was observed for *IL1B* and ApoA1 (ρ = 0.454, *p* = 0.013).

**Figure 4:**
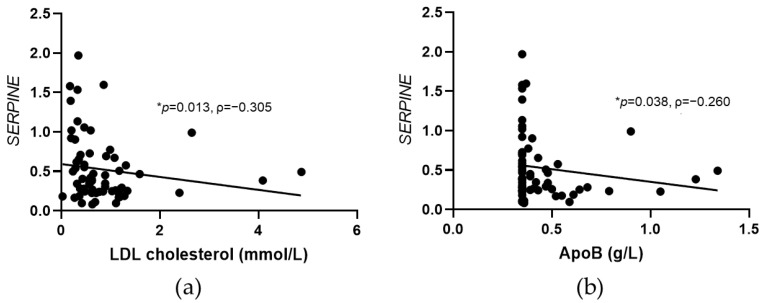
Significant correlations between *SERPINE* expression and lipid parameters in CAD patients following PCSK9 inhibitors treatment. Significant correlations were found between *SERPINE* and LDL cholesterol (**a**) and apolipoprotein B (ApoB) (**b**) in patients with CAD after six months of PCSK9 inhibitors treatment. Scatter plots are shown with the results of Spearman correlation analysis (* *p* < 0.05; ρ, Spearman’s correlation coefficient). Gene expression data are normalized to geometric mean of glyceraldehyde-3-phosphate dehydrogenase (*GAPDH*) and ribosomal protein L13a (*RPL13A*).

In patients with CAD after PCSK9 inhibitor treatment, significant negative correlations were observed for *SERPINE* and LDL cholesterol and ApoB levels (Figure 4).

### 3.4. Treatment with PCSK9 Inhibitors Influences the Expression of VEGFA, F3 and SERPINE

**Figure 5:**
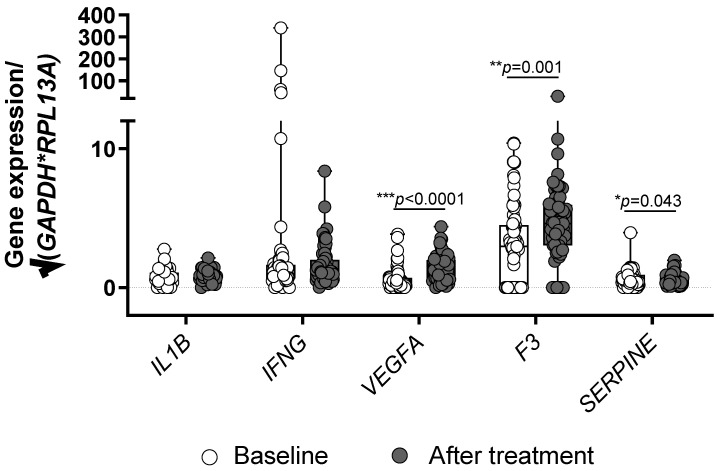
Comparison of the gene expression profile before and after treatment with PCSK9 inhibitors in patients with CAD. Significant differences were obtained for *VEGFA*, *F3* and *SERPINE* as indicated (*** *p* < 0.0001, ** *p* < 0.001, * *p* < 0.05, Wilcoxon signed rank test). Shown are box and whisker plots with individual values. Gene expression data are normalized to geometric mean of glyceraldehyde-3-phosphate dehydrogenase (*GAPDH*) and ribosomal protein L13a (*RPL13A*).

To evaluate the effect of the treatment with PSCK9 inhibitors on gene expression of the studied markers in patients with CAD, the data on gene expression at baseline and after six months of treatment were compared (Figure 5). Statistically significant differences were observed for *VEGFA* (*p* < 0.0001), *F3* (*p* = 0.001), and *SERPINE* (*p* = 0.043). Treatment with PCSK9 inhibitors increased the expression of *VEGFA* and *F3* encoding TF, and decreased *SERPINE* encoding PAI-1. No differences were observed for *IL1B* and *IFNG* expression (*p* = 0.533 and 0.781).

## 4. Discussion

To our knowledge, the present study is the first to investigate the effect of PCSK9 inhibitors on markers of inflammation, angiogenesis, coagulation and fibrinolysis in patients with stable CAD. Given that all of our patients were treated with the maximum tolerated doses of statins, it is not surprising that they had lower levels of both total and LDL cholesterol and ApoB in comparison with healthy controls. Lp(a) values were extremely low in the control group and extremely high in the patient group, where the Lp(a) inclusion criterion was Lp(a) above 600 mg/L. Compared to healthy peers, patients with stable CAD had significantly lower levels of genes encoding IL-1β and VEGF-A. At a first glance, these results are surprising, as increased gene expression would result in higher concentrations of these risk factors in healthy people than in patients with stable CAD. However, it is more likely that lower IL-1β and VEGF-A levels in CAD patients are the consequence of statin treatment [27]. The same is true for the higher expression of the *F3* gene encoding TF, the main factor that triggers the coagulation process [28], which suggests a higher concentration of TF in healthy controls. However, we must be aware that in addition to statins, all of our patients have been treated for more than six months also with ACE inhibitors/sartans, β blockers, and acetylsalicylic acid. The effect on the parameters of inflammation is well-recognized for statins [16,17], ACE inhibitors/sartans [29], β blockers [30], and acetylsalicylic acid [31], while all of them except β blockers also affect coagulation and fibrinolysis [16,17,29,31]. The inflammatory cytokine IL-1β stimulates the endothelial cells and leukocytes to produce intracellular adhesion molecule-1 (ICAM-1) and vascular cell adhesion molecule-1 (VCAM-1) [6]. Increased levels of VEGF-A in patients with ischemic heart disease lead to neovascularization of atherosclerotic plaques and thus make them more prone to rupture and consequently to arterial thrombosis [32]. However, VEGF-A, on the other hand, also has positive effects as it protects the endothelial cells by increasing the expression of anti-apoptotic proteins and nitric oxide [33]. Atherosclerotic lesions contain a large amount of TF, which is released upon rupture and thus causes thrombosis and the consequent acute cardiovascular event [9]. Moreover, we have found significant negative correlation of IL-1β gene expression with HDL cholesterol and a positive correlation with triglycerides in healthy adults. Similar results have been previously observed by Teixeira Sliva et al. in young adolescent population [34]. The negative association between IL-1β and HDL cholesterol concentration found in our healthy control group is not surprising, as HDL cholesterol is known to have anti-inflammatory effects [35]. The anti-inflammatory properties of HDL cholesterol may account for at least part of the anti-atherogenic potential of these lipoproteins. Anti-inflammatory properties of HDL cholesterol are superior to HDL cholesterol concentration in terms of discriminating between those subjects with and without CAD. Given that the concentration of triglycerides and HDL cholesterol is inversely proportional [36], a positive correlation between the triglycerides and *IL1B*, and a negative correlation between HDL cholesterol and *IL1B* found in our study, seem reasonable.

Lp(a) is susceptible to oxidative modifications and the formation of pro-inflammatory and pro-atherogenic oxidized phospholipids [37]. Lp(a) carries more than 80% oxidized phospholipids in its particles, and consequently this increases the inflammatory activity of the arterial wall [38]. Treatment with PCSK9 inhibitors in patients with CAD included in our study resulted in a 63% reduction in LDL cholesterol and a 21% reduction in Lp(a). Since the decrease in both LDL and Lp(a) cholesterol levels was not associated with a change in the expression of the studied genes in our study (data not shown), we cannot assess whether these changes are related to the lipolytic effect of the PCSK9 inhibitors or the so-called pleotropic effects. In our study, we did not detect any change in the IL-1β gene expression after PCSK9 treatment. This finding may be related to the results of the previous studies that did not detect any changes in hsCRP, an indicator of systemic inflammation, in patients treated with PCSK9 inhibitors, regardless of whether they were previously treated with statins or not [39,40]. In addition to the systemic inflammatory response, local inflammatory events at the site of atherosclerotic bed as well as in the bed itself are also important in all phases of the atherosclerotic process. In patients with elevated LDL and Lp(a) levels treated with statins, additional treatment with the PCSK9 inhibitor evolocumab did not alter either local inflammation in the arterial wall or systemic inflammation [40]. On the contrary, in patients with CAD or familial hypercholesterolemia not treated with statins due to statin intolerance, treatment with alirocumab attenuated arterial wall inflammation without changing systemic hsCRP [39]. The only difference between the two studies was the higher Lp(a) concentration at both the baseline and the end of the first study. Marques et al. recently evaluated the effects of PCSK9 inhibition on inflammatory state, endothelial dysfunction and cardiovascular outcomes in patients with familiar hypercholesterolemia treated with maximum statin doses and, if necessary, with ezetimibe [41]. They showed that eight weeks of alirocumab treatment decreased INF-γ and increased IL-10 levels, while it had no effect on the concentration of IL-6 and TNF-α [41]. The main difference between the study of Marques et al. and our study is that we have included patients after an acute cardiovascular event. However, their study also does not include data on the concentration of Lp(a), which is an important factor involved in inflammatory events in the process of atherosclerosis [42].

Given the pro- and anti-atherosclerotic effects of VEGF-A, it is difficult to evaluate whether an increase in its concentration after treatment with PCSK9 inhibitors contributes to a reduction in the incidence of acute cardiovascular events, as found in large randomized double-blind studies with both alirocumab [2] and evolocumab [43]. To determine whether increased *F3* expression leads to increased TF concentration after treatment with PCSK9 inhibitors, increasing the chance of activating the coagulation cascade, a study measuring both *F3* gene expression and TF concentration would be needed. In prospective and case-control studies, PAI-1 levels were found to be associated with the risk of first [44] and recurrent acute myocardial infarction [45]. All of our patients have been previously treated with statins that reduce PAI-1 levels and activity in patients with known ischemic heart disease, regardless of the statin type, dose, and duration of treatment [46]. Given that there was a decrease in *SERPINE* expression in our patients after treatment with PCSK9 inhibitors, it could be assumed that this is one of the mechanisms that helps to reduce PAI-1 antigen and consequently improve fibrinolytic activity. We have also found negative correlations between *SERPINE* expression and LDL cholesterol and ApoB levels in CAD patients after PCSK9 inhibitors treatment. In patients with isolated hypercholesterolemia previously not treated with any hypolipidemic drugs treatment with alirocumab decreased PAI-1 antigen, F VII activity and fibrinogen concentration [47].

There are also limitations to our study. The main one is that the data on markers of inflammation, angiogenesis, coagulation and fibrinolysis were obtained at the gene expression level only, hence further validation on the protein level is required to establish a pathophysiological role of these markers in CAD. However, the advantage of our study is that it had an adequate number of patients as well as the inclusion of healthy controls to be able to detect the differences in the studied genes.

## 5. Conclusions

Beside lipids, inflammation, angiogenesis, coagulation and fibrinolysis play very important roles in all stages of the atherosclerotic process. Drugs from the PCSK9 inhibitor group reduce the incidence of cardiovascular morbidity and mortality in patients with known cardiovascular disease. Most of this beneficial effect is due to a decrease in LDL cholesterol and Lp(a), but probably at least part of this is due to influence on non-lipid risk factors such as the effects on inflammation, angiogenesis, coagulation and fibrinolysis. Our results underpin the previous knowledge by demonstrating the importance of the genes encoding these markers, in particular IL-1β, VEGF-A and TF in CAD patients with severely elevated Lp(a) values and not reaching the target LDL cholesterol levels. Moreover, we also showed the beneficial effect of PCSK9 treatment in these patients on the expression of the genes involved in the inflammatory process, angiogenesis and fibrinolytic activity. To determine whether these effects are also clinically relevant, a randomized double-blind study would be needed to monitor the inflammatory and coagulation and fibrinolysis parameters at both the genetic and protein levels in addition to the effects on the occurrence of new cardiovascular events. However, to determine the extent to which these effects are dependent on a decrease in Lp(a), a similarly designed study would be needed in patients with low LDL and high Lp(a) cholesterol treated with drugs specific to decrease Lp(a).

## Figures and Tables

**Table 1:** Baseline characteristics of the three study groups.

	Controls (N = 27)	Placebo (N = 30)	Treated (N = 67)	*p* (Controls vs. Placebo)	*p* (Controls vs. Treated)
Age at inclusion (years)	51 (47–52)	49 (43–55)	53 (46–56)	0.108	
M/F	21/6	29/1	58/9	0.103	
Total cholesterol (mmol/L)	5.72 (5.22–6.18)	4.08 (3.55–4.84)	4.24 (3.69–4.74)	*** < 0.0001	*** < 0.0001
HDL cholesterol (mmol/L)	1.37 (1.20–1.87)	1.05 (0.95–1.31)	1.15 (1.00–1.32)	*** < 0.0001	* 0.003
LDL cholesterol (mmol/L)	3.40 (3.18–4.00)	2.31 (1.70–2.69)	2.34 (1.73–2.65)	*** < 0.0001	*** < 0.0001
Triglycerides (mmol/L)	1.32 (1.01–2.03)	1.64 (1.02–2.20)	1.41 (1.05–2.13)	0.853	
Lp(a) (mg/L)	11.0 (4.0–18.0)	1477 (1113–1702)	1400 (1187–1664)	*** < 0.0001	*** < 0.0001
ApoA1 (g/L)	1.14 (1.03–1.26)	0.79 (0.73–0.985)	0.82 (0.63–0.97)	* 0.004	0.054
ApoB (g/L)	1.42 (1.29–1.58)	1.23 (1.16–1.35)	1.30 (1.20–1.46)	*** < 0.0001	*** < 0.0001

M/F, male/female; HDL cholesterol, high density lipoprotein cholesterol; LDL cholesterol, low density lipoprotein cholesterol; Lp(a), lipoprotein(a); apo, apolipoprotein. Data are presented as medians (25–75%). Differences between the controls and the placebo groups (Controls vs. Placebo) and controls and PCSK9 inhibitor treated groups (Controls vs. Treated) were observed as indicated (*** *p* < 0.0001, * *p* < 0.05, Kruskal-Wallis test with Bonferroni multiple comparison tests).

## Data Availability

The data presented in this study are available upon request from the corresponding author.

## Supplementary Materials

The following supporting information can be downloaded at: www.mdpi.com/article/10.3390/jcdd9070211/s1, Table S1: Primer pair sequences used for the gene expression profiling.

## Abbreviations

ApoA1	apolipoprotein A1
ApoB	apolipoprotein B
CAD	coronary artery disease
HDL cholesterol	high density lipoprotein cholesterol
hsCRP	high sensitivity C reactive protein
IFN-γ	interferon-γ
IL-1β	Interleukin-1β
LDL cholesterol	low density lipoprotein cholesterol
Lp(a)	lipoprotein(a)
PAI-1	tissue plasminogen activator inhibitor-1
PCSK9	proprotein convertase subtilisin/kexin type 9
TF	tissue factor
VEGF-A	vascular endothelial growth factor-A
