# Supplementary material for: Gene Expression Profiling of Markers of Inflammation, Angiogenesis, Coagulation and Fibrinolysis in Patients with Coronary Artery Disease with Very High Lipoprotein(a) Levels Treated with PCSK9 Inhibitors
Source: J Cardiovasc Dev Dis. 2022 Jul 1;9(7):211. doi: 10.3390/jcdd9070211 (PMC9324258; doi:10.3390/jcdd9070211)
Supplement: Supplementary file 1 [file jcdd-09-00211-s001.zip › jcdd-1771143-supplementary.pdf]

Supplementary Table.

Supplementary Table S1. Primer pair sequences used for the gene expression profiling.

Gene symbol	Forward primer (5'-3')	Reverse primer (3'-5')	Reference
IL1B	ACGATGCACCTGTACGAT	AGAACACCACTTGTTGCTCCAT	[23]
IFNG	ATTGGAAAGAGGAGAGTGACAGA	CACACTCTTTTGGATGCTCTGG	[23]
VEGFA	AGCCTTGCCTTGCTGCTCTACC	GTGATGATTCTGCCCTCCTCCTTC	[24]
F3	CAGAGTTCACACCTTACCTGGAG	GTTGTTCCTTCTGACTAAAGTCCG	[26]
SERPINE	CTCATCAGCCACTGGAAAGGCA	GACTCGTGAAGTCAGCCTGAAAC	[25]
GAPDH	TGCACCACCAACTGCTTAGC	TGGCATGGACTGTGGTCATG	[23,24]
RPL13A	AAAAAGCGGATGGTGGTTC	CTTCCGGTAGTGGATCTTGG	[22]

IL1B, interleukin 1β gene; *IFNG*, interferon-γ gene; *VEGFA*, vascular endothelial growth factor-A gene; *F3*, tissue factor gene; *SERPINE*, plasminogen activator inhibitor-1 gene; *GAPDH*, glyceraldehyde-3-phosphate dehydrogenase gene; *RPL13A*, ribosomal protein L13a gene.
